# Oil yield prediction for sunflower hybrid selection using different machine learning algorithms

**DOI:** 10.1038/s41598-023-44999-3

**Published:** 2023-10-17

**Authors:** Sandra Cvejić, Olivera Hrnjaković, Milan Jocković, Aleksandar Kupusinac, Ksenija Doroslovački, Sonja Gvozdenac, Siniša Jocić, Dragana Miladinović

**Affiliations:** 1 Institute of Field and Vegetable Crops, Novi Sad, Serbia; 2 Faculty of Technical Sciences, University of Novi Sad, Novi Sad, Serbia

**Keywords:** Computational biology and bioinformatics, Plant sciences

## Abstract

Due to the increased demand for sunflower production, its breeding assignment is the intensification of the development of highly productive oil seed hybrids to satisfy the edible oil industry. Sunflower Oil Yield Prediction (SOYP) can help breeders to identify desirable new hybrids with high oil yield and their characteristics using machine learning (ML) algorithms. In this study, we developed ML models to predict oil yield using two sets of features. Moreover, we evaluated the most relevant features for accurate SOYP. ML algorithms that were used and compared were Artificial Neural Network (ANN), Support Vector Regression, K-Nearest Neighbour, and Random Forest Regressor (RFR). The dataset consisted of samples for 1250 hybrids of which 70% were randomly selected and were used to train the model and 30% were used to test the model and assess its performance. Employing MAE, MSE, RMSE and R2 evaluation metrics, RFR consistently outperformed in all datasets, achieving a peak of 0.92 for R2 in 2019. In contrast, ANN recorded the lowest MAE, reaching 65 in 2018 The paper revealed that in addition to seed yield, the following characteristics of hybrids were important for SOYP: resistance to broomrape (*Or*) and downy mildew (*Pl*) and maturity. It was also disclosed that the locality feature could be used for the estimation of sunflower oil yield but it is highly dependable on weather conditions that affect the oil content and seed yield. Up to our knowledge, this is the first study in which ML was used for sunflower oil yield prediction. The obtained results indicate that ML has great potential for application in oil yield prediction, but also selection of parental lines for hybrid production, RFR algorithm was found to be the most effective and along with locality feature is going to be further evaluated as an alternative method for genotypic selection.

## Introduction

Sunflower (*Helianthus annuus* L.) is an important oil crop, occupying more than 28 million hectares worldwide^1^. The overall world market trend of increasing sunflower production from year to year, and in the 2021/2022 season it is expects overall world production to grow by around 16%^2^. Sunflower has been recognized as a significant source of high-quality edible oil for human consumption^3^. Consumption of edible vegetable oils on a global scale has steadily increased from 87.8 million tons in the year 2000 to 186.5 million tons in 2016^4^. Therefore, the sunflower breeding assignment is intensifying the development of highly productive oil seed hybrids to satisfy the demand for the oil edible industry. Generally, hybrids are created by making crosses of two parental lines. Whether these crosses produce better hybrids (e.g., with higher seed and oil yields and better adaptability to regional soil and weather conditions) depends on the combining ability performance^5^. Newly developed hybrids need to be tested in different environments. The adaptation of the sunflower to different climatic and soil conditions has enhanced its cultivation as an oilseed crop worldwide^6^.

Global climate change is challenging for sunflower breeders due to the time development of hybrids and it can take 10–15 years. Therefore, breeders must develop hybrids for certain and uncertain environmental changes from one decade into the future based on field trials conducted in the current weather conditions^7^. Selection decisions for yield traits with significant genotype-environment interactions (GEI) can be partially controlled using a stress-controlled environment and adequate environmental characterization. Accurate yield prediction helps breeders manage decisions and support plant strategies and breeding programs for different purposes. Yield predictions have been generated by statistical and mathematical models, which generally have complementary strengths and limitations.

One of the most significant objectives in precision agriculture is to improve crop yield production and quality while minimizing expenses. Early sunflower oil yield estimation can help the selection process by shortening the breeding plans as a quantitative trait sunflower oil yield depends on various features such as hybrid characteristics, weather, soil properties and other features. The need to discover these inputs has led to increased adoption of remote and proximal sensing technologies^8^ in precision agriculture^9^. Recently, a few of scientific literature has emerged, focusing on utilizing ML algorithms to predict the performance of sunflower crops. Predominantly, the application of machine learning models was focused on the prediction of yield outcomes^10,11^, as well as the prediction of traits encompassing disease identification and resistance^12,13^, seed quality assessments^14,15^, thereby facilitating the selection of superior sunflower hybrids. Furthermore, machine learning models were also used to analyze images and remote sensing of sunflower plants for various traits^16–18^. Most of the mentioned work used metadata to build prediction models based on various parameters such as weather conditions, soil properties, and management practices to predict yield or detect and diagnose diseases in sunflower plants based on visual symptoms or sensor data, aiding in timely disease management. In contrast to these research efforts, the novelty of our research is using of empirical data for predicting oil yield, involving the characteristic and measured data of experimental sunflower hybrids aiming to select superior sunflower hybrids resilient to different climate conditions.

The application of any particular modelling approach depends on the goal of prediction and relevant constraints. We aimed to use a two-year dataset for Sunflower Oil Yield Prediction (SOYP) of potential new hybrids, which would benefit sunflower breeders in identifying desirable new hybrids with high oil yield.

The main goals of this paper are as follows:

- To evaluate the possibility of using ML for prediction sunflower oil yield and selection of parental lines in hybrid production based on datasets containing newly developed sunflower hybrids tested in trials in 2018 and 2019.
- To investigate and compare ML models’ performances and select the most effective one.
- To determine the essential features affecting oil yield prediction to fasten the breeding process and increase accuracy in selecting the most desirable sunflower hybrids.

## Results and discussion

### Models’ performance

**Figure 1:**
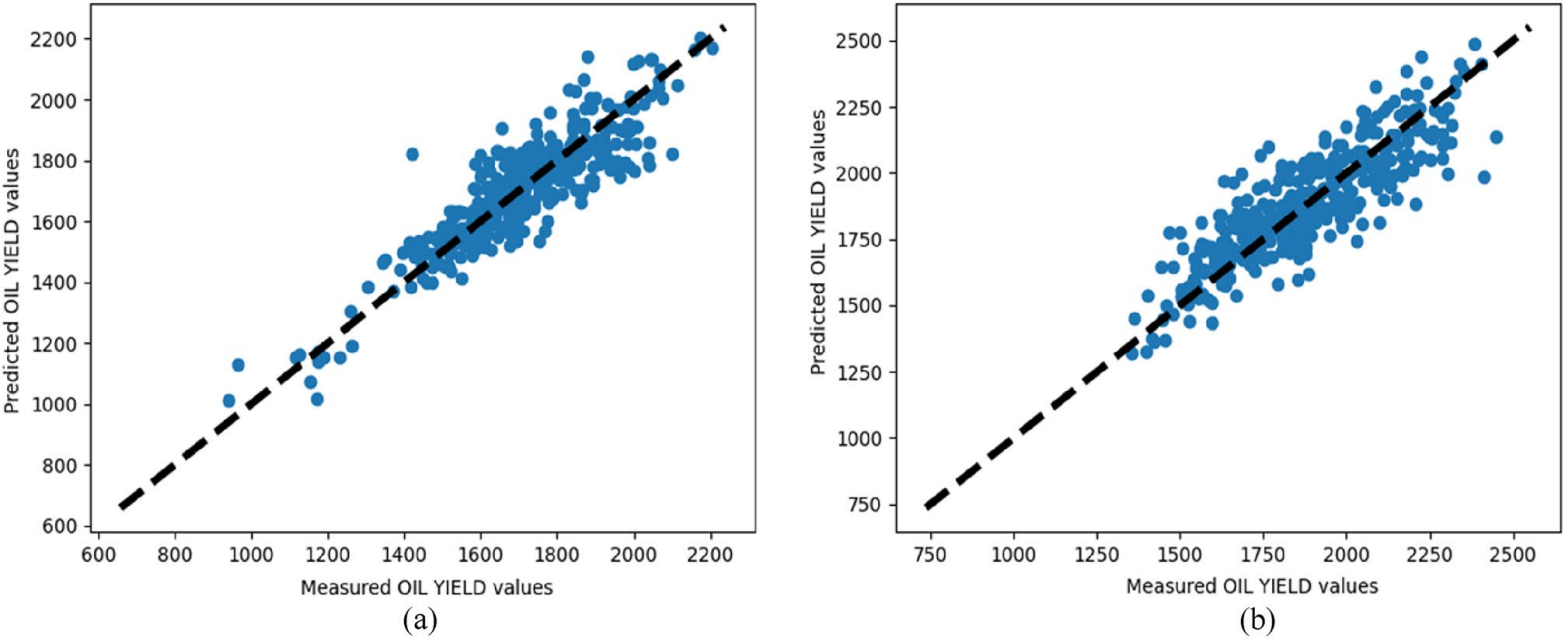
Measured and predicted oil yield values for the first data subset for the RFR in (**a**) 2018 and (**b**) 2019.

**Figure 2:**
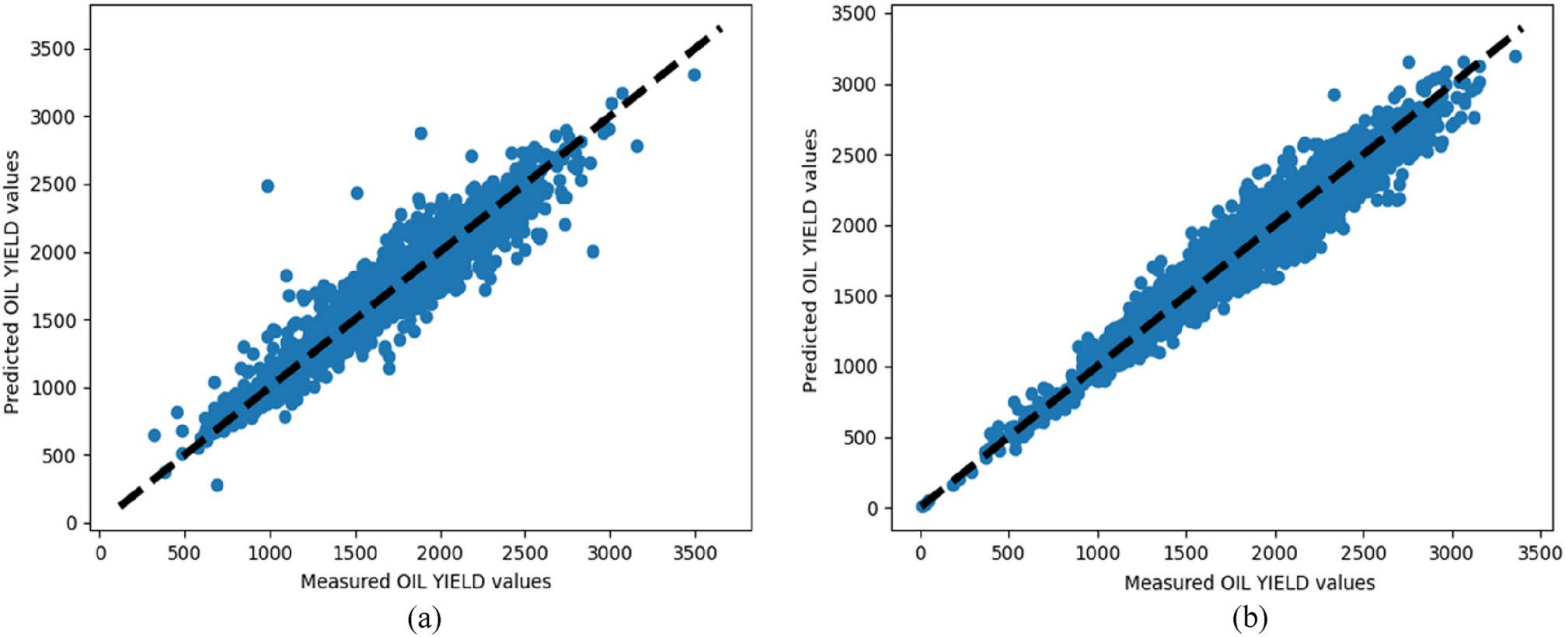
Measured and predicted oil yield values for the second data subset for the RFR in (**a**) 2018 and (**b**) 2019.

Models for sunflower oil yield prediction were implemented with RFR, KNN, ANN and SVR algorithms using features from two data subsets for the 2018 and 2019 year. (Tables 1 and 2). For almost all model evaluation criteria the RFR algorithm achieved the best results for both years as shown in Figs. 1 and 2. ANN yielded best results only for MAE in 2018. Moreover, ANN yielded second best results for the first data subset and KNN algorithm produced the second-best results for the second data subset.

**Table 1:** Results for the first data subset.

Year	Algorithm	Training errors				Test errors			
		MAE	MSE	RMSE	R2	MAE	MSE	RMSE	R2
2018	Support vector regression	144	36,486	191	0.11	138	33,140	182	0.11
	Random forest regression	25	1253	35	0.96	69	**8295**	**91**	**0.78**
	K-nearest neighbours	69	10,333	101	0.75	70	9491	97	0.75
	Neural network	70	10,353	101	0.75	**65**	8826	93	0.76
2019	Support vector regression	167	45,201	212	0.06	176	47,096	217	0.07
	Random forest regression	36	2272	47	0.95	**95**	**14,606**	**120**	**0.71**
	K-nearest neighbours	110	18,574	136	0.61	117	20,466	143	0.6
	Neural network	117	20,466	143	0.60	112	19,137	138	0.62

Significant values are in bold.

**Table 2:** Results for the second data subset.

Year	Algorithm	Training errors				Test errors			
		MAE	MSE	RMSE	R2	MAE	MSE	RMSE	R2
2018	Support vector regression	113	26,026	161	0.83	112	24,037	155	0.83
	Random forest regression	31	2024	44	0.98	**81**	**13,036**	**114**	**0.91**
	K-nearest neighbours	90	15,637	125	0.9	91	15,783	125	0.89
	Neural network	99	19,517	139	0.87	98	18,662	136	0.87
2019	Support vector regression	157	42,586	206	0.75	160	43,607	208	0.75
	Random forest regression	32	1880	43	0.98	**86**	**12,918**	**113**	**0.92**
	K-nearest neighbours	104	18,255	135	0.89	110	20,392	142	0.88
	Neural network	128	25,854	160	0.85	131	26,870	163	0.84

Significant values are in bold.

When comparing the R2 values of prediction algorithms with each feature subsets, the RFR algorithm took first place. RFR has a 0.78, KNN has a 0.75, ANN has a 0.76, SVR has a 0.11 for the first data subset in 2018. Similar values were obtained for the first data subset in 2019 where RFR, KNN, ANN, and SVR have scores of 0.71, 0.6, 0.62 and 0.07 respectively. For the second data subset in 2018, RFR, KNN, ANN and SVR all have 0.91, 0.89, 0.87, and 0.83 respectively. RFR, KNN, ANN, and SVR each have 0.92, 0.88, 0.84, and 0.75 for the second data subset in 2019. RFR is a good prediction algorithm based on the overall performance and taking distinct feature subsets into account, KNN and ANN provide good accuracy.

### Feature importance and statistical analysis

Feature importance assigns a score to input features based on how useful they are at predicting a target variable. Feature importance is significant because it provides insight into the data and the model. In our study, we utilized the random forest regressor, a tree-based ensemble learning method. Within this algorithm, feature importance is derived from the decrease in node impurity, which is averaged over all the decision trees in the forest. More specifically, the impurity decrease from each feature is computed as the difference between the impurity of a node and the weighted sum of the impurities of its child nodes. When a feature consistently results in nodes with high purity (low impurity), it's deemed important. In other words, features that more frequently split nodes in ways that reduce impurity tend to be more relevant for predictions. After training the random forest regressor, we retrieve the feature importance via the model's feature_importance attribute. We then visualize these importance using a horizontal bar chart, which clearly delineates the relative significance of each feature in our dataset.

**Figure 3:**
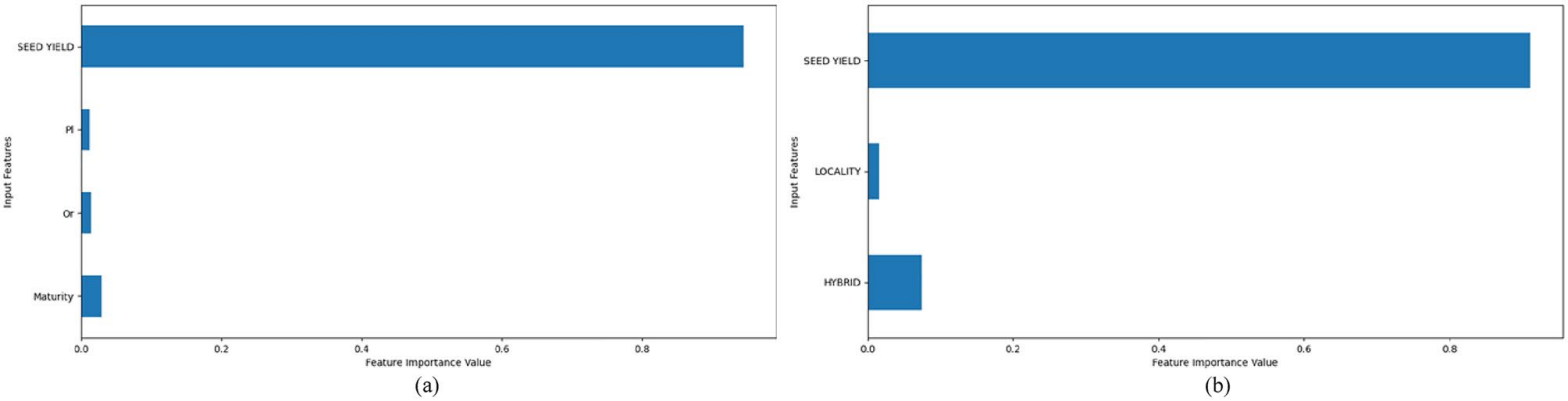
Important features in RFR for 2018 (**a**) first data subset and (**b**) second data subset.

**Figure 4:**
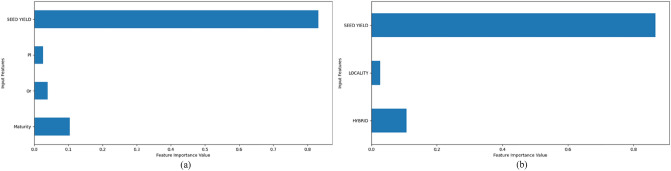
Important features in RFR for 2019 (**a**) first data subset and (**b**) second data subset.

Figures 3 and 4 show the importance of the selected most relevant features. For the first data subset, we chose four most important features and for the second we chose two (because of the number of predictor features). We used a feature importance property which the model provides after being fit and which can be used to retrieve the relative importance scores for each input feature. When it comes to the first subset of data, for both years it was determined that the most relevant features were seed yield, *Pl*, *Or*, and Maturity. When it comes to the second subset of data, in addition to the seed yield, the feature that describes the used hybrid was also important. This applies to both years as well.

**Figure 5:**
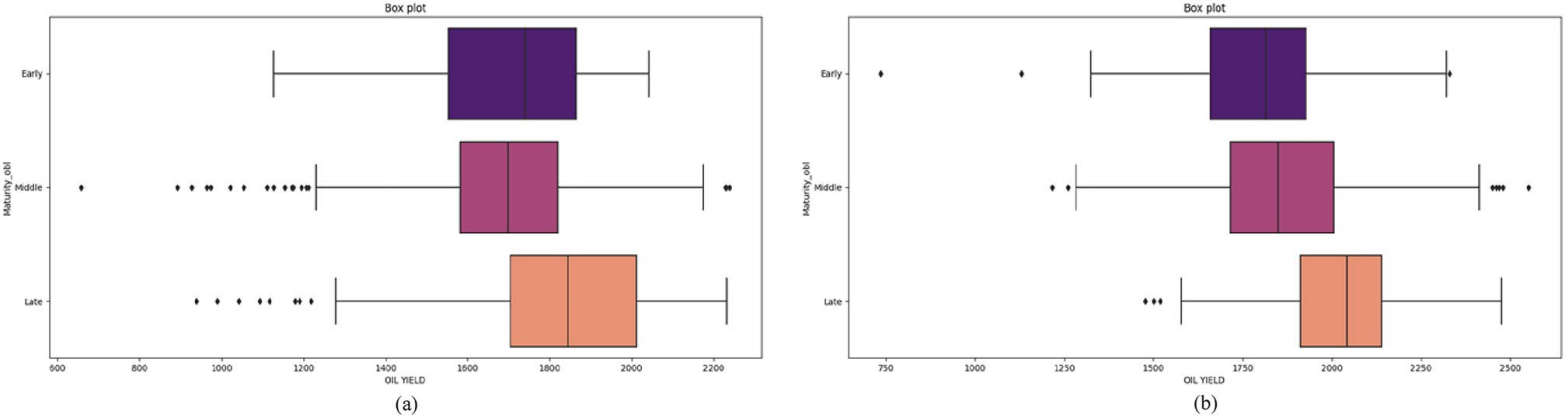
Oil yield values for maturity feature in (**a**) 2018 and (**b**) 2019.

**Figure 6:**
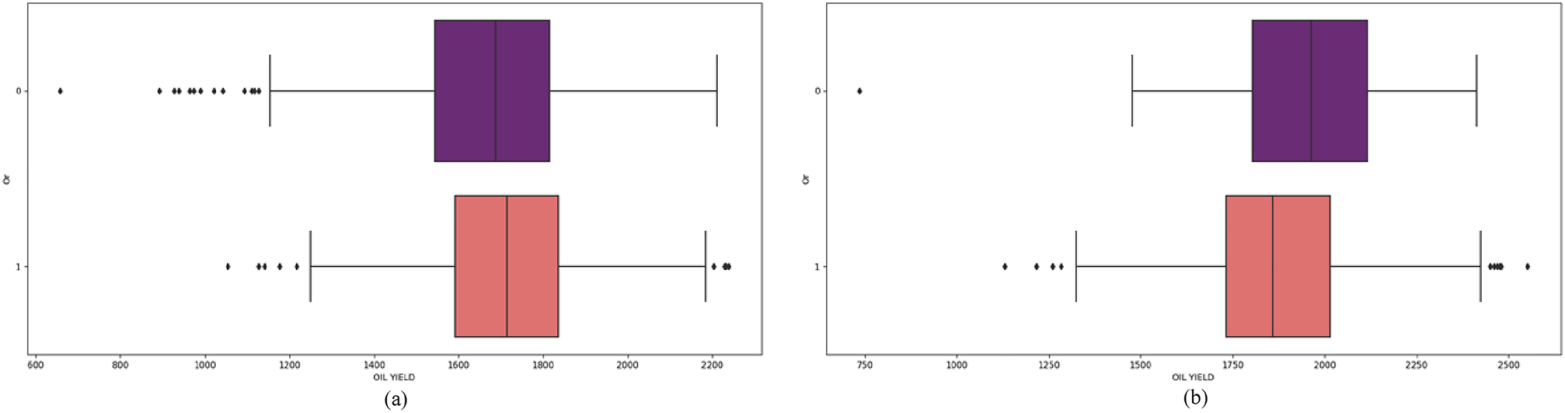
Oil yield values for *Or* feature in (**a**) 2018 and (**b**) 2019.

**Figure 7:**
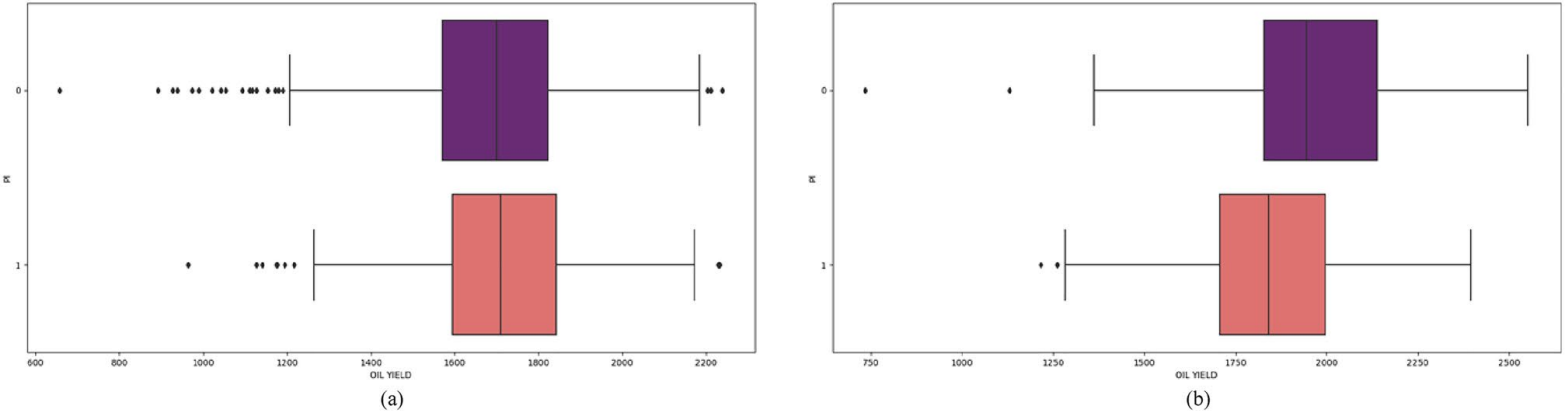
Oil yield values for *Pl* feature in (**a**) 2018 and (**b**) 2019.

Statistical analysis can efficiently establish the relationships between various features. One of the main contributions of this work is to establish a relationship between oil yield, environmental conditions, and characteristics of hybrids. Figures 5, 6, and 7 show oil yield values in relation to detected important features.

Maturity had a significant contribution to the prediction of sunflower oil yield. As expected, late-maturity hybrids had higher oil yield performance than middle and early hybrids. Interestingly, early hybrids in 2018 had a more comprehensive range of performance, showing that some early hybrids have a high genetic potential for oil yield, suggesting further investigation. Although higher temperatures and less rainfall in 2018, the oil yield performance of examining hybrids was higher in 2019. The distribution of rain in 2019 was more favourable for sunflower production depending on the sunflower growth phase and water uptake.

Resistance to broomrape (*Or*) and downy mildew (*Pl*) are important features in sunflower production. Resistant hybrids had higher oil yield in 2018 while sensitive in 2019. In 2019 hybrid performance of oil yield was more elevated, recommending a more significant prediction of oil yield. Incorporating resistance genes into sunflower hybrids, such as *Or* and *Pl*, other traits that affect the reduction of seed and oil yields are often introduced^19^. It takes several cycles of crossbreeding to eliminate negative characteristics. The existence of materials in earlier cycles still needs to be improved in terms of oil yields.

**Figure 8:**
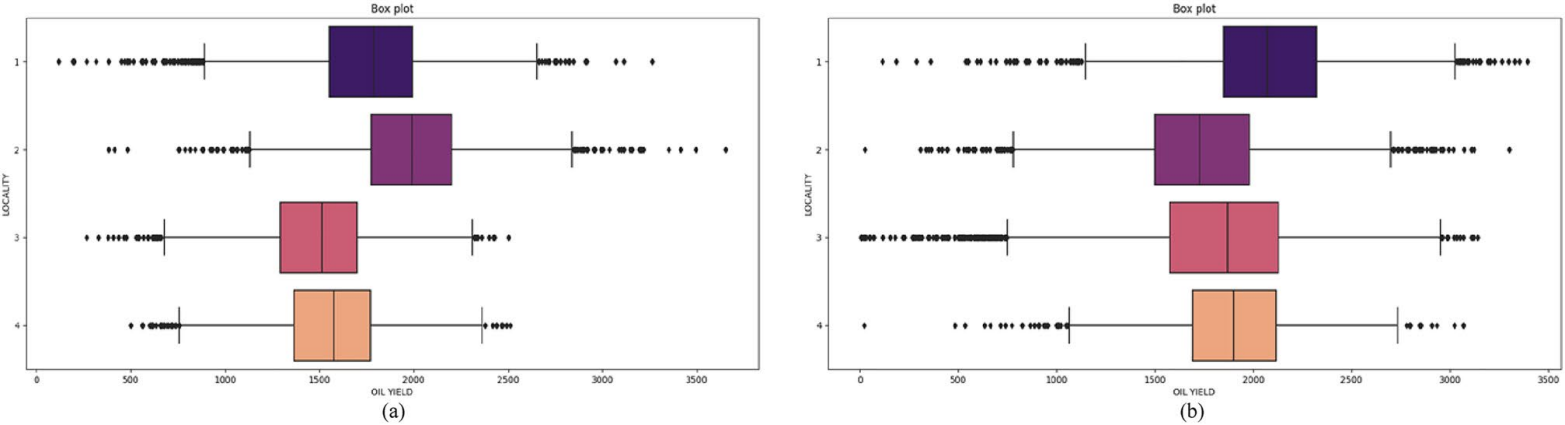
Oil yield values for 4 localities in (**a**) 2018 and (**b**) 2019.

It is interesting to compare the oil yields at each of the locations. Figure 8 shows box plots with oil yield values for four localities. From the figure it is seen that the 2019 oil yield values were averagely higher than in the 2018. For the 2018, the oil yield was the highest at the Novi Sad locality, and for the 2019, the oil yield was the highest at the Subotica locality. Oil yield values were similar for localities Kikinda and Vršac in 2018 year. On contrary, all localities except the locality Subotica in the 2019 have similar oil yield. As previously mentioned, locations are geographically close but vary in soil type and microclimate. Based on oil yield results only locality Subotica showed stability of examined hybrids indicating best adaptation to sandy soils while rain and temperature had less impact. However, sandy soil suffers from a fast water deficit which force sunflower to adapt by building deeper root system^20^.

Based on mathematical and statistical analysis some candidate hybrids were distinguished. Among 1250 sunflower hybrid combinations tested in two-year trials, certain have been selected for the highest oil yield performance. Hybrids were created by crossing mother and father lines and that could affect oil content performance. The mother lines have a unique effect since their entire cytoplasm was inherited^21^. Therefore, the tested hybrids could be classified based on the mother line performance. Tables 3 and 4 show hybrids having the highest oil yield. Two mother lines dominated the selected hybrids: the late DF-AB-2 in 2018 and the medium HA-26 in 2019. Combinations with the DF-AB-2 mother gave higher oil yields in the year when there were more elevated temperatures, considering that hybrids with longer growing seasons tolerate dry intervals more easily^22^. Line HA-26 has been the most dominated line in IFVCNS sunflower breeding program and has various isogenic lines^23^.

**Table 3:** Hybrids with the highest oil yield from 2018.

Hybrid	Oil yield (kg ha^−1^)	Or	Pl	Maturity	Description
998	2238.06	1	0	Late	Hybrid based on DF-AB-2 mother
363	2231.15	1	1	Late	Hybrid based on DF-AB-2 mother
773	2230.73	1	1	Middle	Check
798	2228.16	1	1	Middle	Check
139	2211.46	0	0	Late	Hybrid based on DF-AB-2 mother
792	2203.33	1	0	Late	Hybrid based on KINA-B-5 mother
162	2183.30	1	0	Late	Hybrid based on DF-AB-2 mother
372	2179.68	0	0	Late	Hybrid based on DF-AB-2 mother
890	2175.02	0	0	Late	Hybrid based on KINA-B-5 mother
760	2172.36	1	0	Middle	Hybrid based on IMI-AB-12 mother

**Table 4:** Hybrids with the highest oil yield from 2018.

Hybrid	Oil yield (kg ha^−1^)	Or	Pl	Maturity	Description
848	2551.85	1	0	Middle	Hybrid based on HA-26 mother
116	2480.18	1	0	Middle	Hybrid based on HA-26 mother
550	2475.18	1	0	Late	Hybrid based on DF-AB-2 mother
114	2469.05	1	0	Middle	Hybrid based on HA-26 mother
773	2460.96	1	0	Middle	Check
112	2450.10	1	0	Middle	Hybrid based on HA-26 mother
350	2424.00	1	0	Late	Hybrid based on DF-AB-2 mother
229	2414.04	0	0	Middle	Hybrid based on HA-26 mother
325	2403.94	1	0	Late	Hybrid based on DF-AB-2 mother
873	2395.66	1	0	Middle	Check

### The potential of machine learning

Applying machine learning (ML) has opened up new possibilities for creating innovative methods to extract more information due to advances in computing power^24^. Previous similar studies have shown that the ensemble algorithms achieved the best results when it comes to performance. In our study, RFR, an ensemble learning model, achieved the best result consistent with previous studies. The algorithm showed the best results for the data for two years. That demonstrates the trustworthiness of the ensemble approach. In their study, Morales and Vilalobos^25^, also found that RFR algorithm had a better performance than artificial neural networks and regularized linear models in yield prediction in sunflower and wheat and was also easier to execute. Amankulova et al.^17^ also found RFR to be very effective for sunflower crop yield prediction using satellite-derived vegetation indices and crop phenology.

Increased oil yield is one of the most important parameters in sunflower breeding and production. Hence, different methods have been used and models developed for prediction of sunflower oil yield in different crossings, as well as in different environments^26–29^. In recent years, machine learning started to be used in crop-section applications, including yield prediction, disease detection, weed detection, crop quality, and species recognition. Up to our knowledge, this is the first time that different ML algorithms were applied to predict sunflower oil yield, involving the characteristic and measured data of 1250 hybrids. Furthermore, many studies have applied machine learning to predict the yields of different crops using available datasets from published or historical data to build and assess crop yield prediction models for different crops and locations. In contrast to them, in this study, the empirical data were used to conduct and derive predictive insights for breeding selection.

In this study, in addition to seed yield, the presence of *Pl* and *Or* genes and the maturity of hybrids showed low but significant importance for SOYP. Generally, resistance to Downey mildew and broomrape has been found to have positive effect on sunflower crop yield^30^. Since those two traits are controlled by a single dominant gene, they are easily transferred into progeny and less dependent on environmental conditions and consequently good feature for ML prediction models. It was also disclosed that the locality feature could be used to estimate sunflower oil yield. Still, it is highly dependable on weather conditions that affect the oil content feature and seed yield. The similar results were obtained by Khan et al.^31^, who used machine learning for the prediction of oil palm yield. This proves that oil and crop yield prediction is still a significant challenge, as it depends on multiple factors such as weather and soil conditions, hybrid, and plant phenotype^32^.

## Conclusions

The main purpose of this study was to test which ML technique is best for prediction of sunflower oil yield while providing insights into the important hybrid and location characteristics for the prediction. Accurate SOYP can greatly optimize the breeding process, making the development of high-yield hybrids more efficient.

For the first data subset using the random forest regression model, the R2 was 0.78 for the dataset from 2018 and 0.71 for the dataset from 2019. For the second data subset using the same model, the R2 was 0.91 for the dataset from 2018 and 0.92 for the dataset from 2019.

This study also aimed at uncovering significant features for SOYP to make it easier to prepare specific hybrids concerning the years and location conditions. The paper revealed that in addition to seed yield, the following characteristics of hybrids are important for SOYP: resistance to broomrape and downy mildew and maturity. It was also disclosed that the locality feature could be used to estimate sunflower oil yield. Still, it depends highly on weather conditions affecting the oil content and seed yield.

There are several ideas for further research. One idea would be to plant hybrids with different soil characteristics in several locations. Another idea would be to add additional hybrid traits like seed traits (thousand seed mass, hectolitre mass, seed set, seed diameters etc.) to predict seed yield.

## Materials and methods

### Plant material and trials

The data were collected in the Pannonian area, where sunflower is the main oil crop. The dataset included records from 1250 sunflower hybrids newly developed at the Institute of Field and Vegetable Crops, Novi Sad, Serbia. Sunflower hybrids were investigated in 4 different locations (Novi Sad-NS, Subotica-SU, Kikinda-KI, Vršac-VŠ) in Serbia in the years 2018 and 2019. These locations were used for new hybrid testing for many years due to environmental differences in soil, rainfalls and temperature, thus creating microclimate conditions for sunflower growing. Weather data were collected by the Republic Hydrometeorological Institute of Serbia^33^. The average seasonal climatic variability of the four tested locations is shown in Table 5. The average T varied from 13.2 °C in April to 24.5 °C in August, indicating that the temperature regime is well within the favourable range for sunflower development. The growing season in 2018 was warmer than in 2019, and the springs were warmer while the summers were equally warm. The amount of precipitation depended on both the year and the locality. The least amount of rain fell in Vršac locality in 2019, and the most in 2018 out of all surveyed localities. In contrast, more rain fell in Subotica in 2019 and less in 2018.

**Table 5:** The average seasonal climatic variability (year 2018 and 2019) of the four tested locations.

Locality	Coordinates	Soil type		Tavr April (°C)	Tavr May (°C)	Tavr June (°C)	Tavr July (°C)	Tavr Aug (°C)	Tavr Sep (°C)	Tavr (°C)	Deviation from long-term Tavr (°C)	No. day with Tmax > 20 °C	No. day with Tmax > 30 °C	No. day with Tmax > 35 °C	No. days with rainfall	Realized vegetation rainfall in mm	Realized vegetation rainfall in %
Subotica (SU)	46° 05ʹ 53ʺ N; 19° 40ʹ 16ʺ E	Sandy soil	2018	16.5	20.4	21.6	23.0	24.3	18.0	20.63	+ 2.1	169	35	0	51	312	93
			2019	13.3	15.0	23.4	23.0	24.0	17.7	19.40	+ 1.2	131	31	1	56	431	129
Novi Sad (NS)	45° 19ʹ 51ʺ N; 19° 50ʹ 59ʺ E	Chernozem	2018	17.2	20.4	21.4	21.9	24.0	18.5	20.57	+ 2.3	173	37	0	51	436	121
			2019	13.5	14.7	23.2	23.3	24.3	18.2	19.53	+ 1.4	126	40	1	52	415	115
Kikinda (KI)	45° 43ʹ 11ʺ N; 20° 18ʹ 07ʺ E	Humogley + Salty soil	2018	16.7	20.6	21.4	22.4	24.2	18.6	20.65	+ 2.1	176	43	0	55	409	124
			2019	13.2	15.1	23.3	22.5	24.5	18.2	19.47	+ 1.3	133	38	3	55	399	121
Vršac (VŠ)	44° 58′ 25" N, 21° 13′ 17" E	Chernozem	2018	17.3	20.3	20.9	22.1	24.1	18.4	20.52	+ 2.0	171	48	0	45	447	115
			2019	13.2	14.8	23.3	22.9	24.5	18.6	19.55	+ 1.0	129	34	2	44	304	78

The experiments were organized in a randomized complete block design with three replications. Basic plots were split in 4 rows with row length of 10 m. Inter-row spacing was 70 cm and intra-row spacing was 25 cm, and standard cultivation practices were applied. Seed yield data were recorded for each plot, on plants from middle rows to avoid edge effect.

### Dataset

The first subset of data was created to focus on hybrids and their characteristics. Due to this, the mean value of 3 replications and the mean of all locations for each hybrid were calculated. Seed yield data were recorded on middle-row plants to avoid the edge effect. The accurate data for seed yield were obtained by measuring the weight of seeds per individual plot through the utilization of the Easy Harvest software, which was interfaced with the Harvest Master HM 800 system. This measurement process was conducted within the Wintersteiger Delta harvester. Seed yield was presented in a kg ha^-1^ on an 11% moisture basis. Hybrids have different types of use: OIL-oil type hybrids; IMI-hybrids resistant to imidazolinone herbicides; SU-hybrids resistant to tribenuron methyl; HO-high-oleic hybrids; CON-confectionery hybrids. Resistance to broomrape-*Orobanche cumana* (*Or*) and downy mildew caused by *Plasmopara halstedii* (*Pl*) was presented by the presence or absence of *Or/Pl* gene(s) in the hybrid. Hybrid maturity implied whether the hybrid was early maturing (up to 105 days of vegetation cycle), medium (105–115 days), or late (more than 115 days). The oil content within intact seeds (with a sample size of 5 g each) was assessed utilizing the nuclear magnetic resonance (NMR) technique employing the Maran Ultra—10 analyser. The results were then quantified as a percentage in relation to the seed's dry weight. The oil yield was calculated from seed yield and oil content presented in kg ha^-1^ (Table 6).

**Table 6:** Description of the first data subset.

Feature	Feature type	Levels	Details
Seed yield (kg ha^−1^)	Predictor	3	Low, medium, high
Hybrid type	Predictor	5	OIL, IMI, SU, HO, CON
Resistance to broomrape	Predictor	2	No *Or* genes, with *Or* gene(s)
Resistance to downy mildew	Predictor	2	No *Pl* genes, with *Pl* gene(s)
Maturity	Predictor	3	Early, medium, late
Seed oil yield	Target	–	–

The second subset of data was created by taking into account all the values of the hybrids at different locations (without using the mean value). Therefore, the hybrid traits could not be used in the second data subset. In this way, the focus was on the characteristics of the locality and its weather conditions presented in Table 5. Data from both years were subjected to the aforementioned procedure (Table 7).

**Table 7:** Description of the second data subset.

Feature	Feature type	Levels	Details
Seed yield (kg ha^−1^)	Predictor	3	Low, medium, high
Locality (weather parameters)	Predictor	4	SU, NS, KI, VŠ
Hybrid	Predictor	1250	Hybrid type
Seed oil yield	Target	–	–

### Exploratory data analysis

Exploratory Data Analysis (EDA) is an approach to summarize the data by identifying its key features and visualize them with proper representations. EDA gives us a visual and numeral data representation by describing the data sets number of rows/columns, missing data, data types, etc. Also, it contains methods to clean corrupted data, and handle missing data, invalid data types, and incorrect values^34^.

There were no missing values in the dataset from 2018, which included 1250 hybrids for 3 replications for each of the 4 localities, with a total of 15,000 records. After eliminating missing values, dataset from 2019 consisted of 14,699 records.

**Figure 9:**
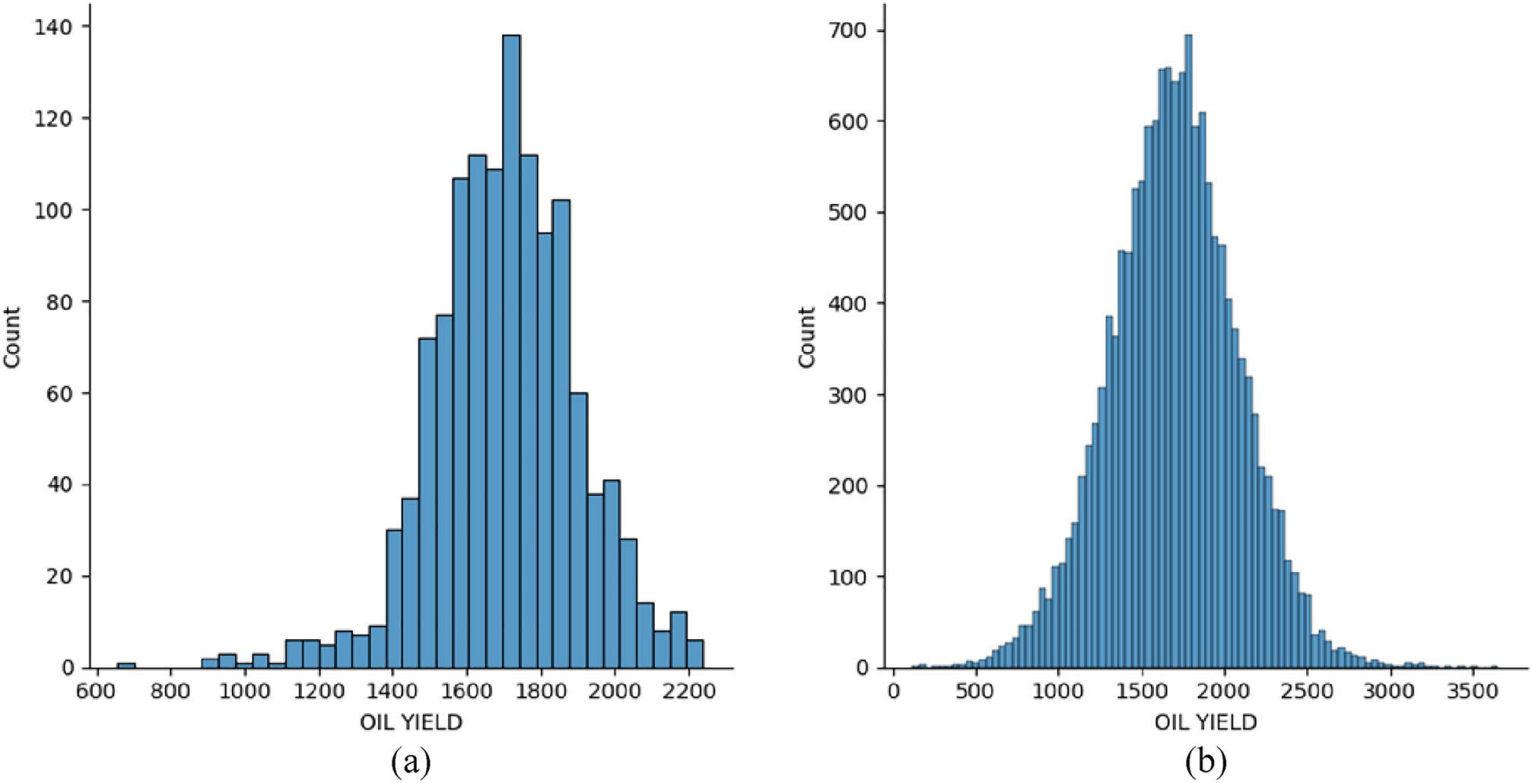
Target value (oil yield) distribution for the data from 2018 (**a**) first data subset (**b**) second data subset.

**Figure 10:**
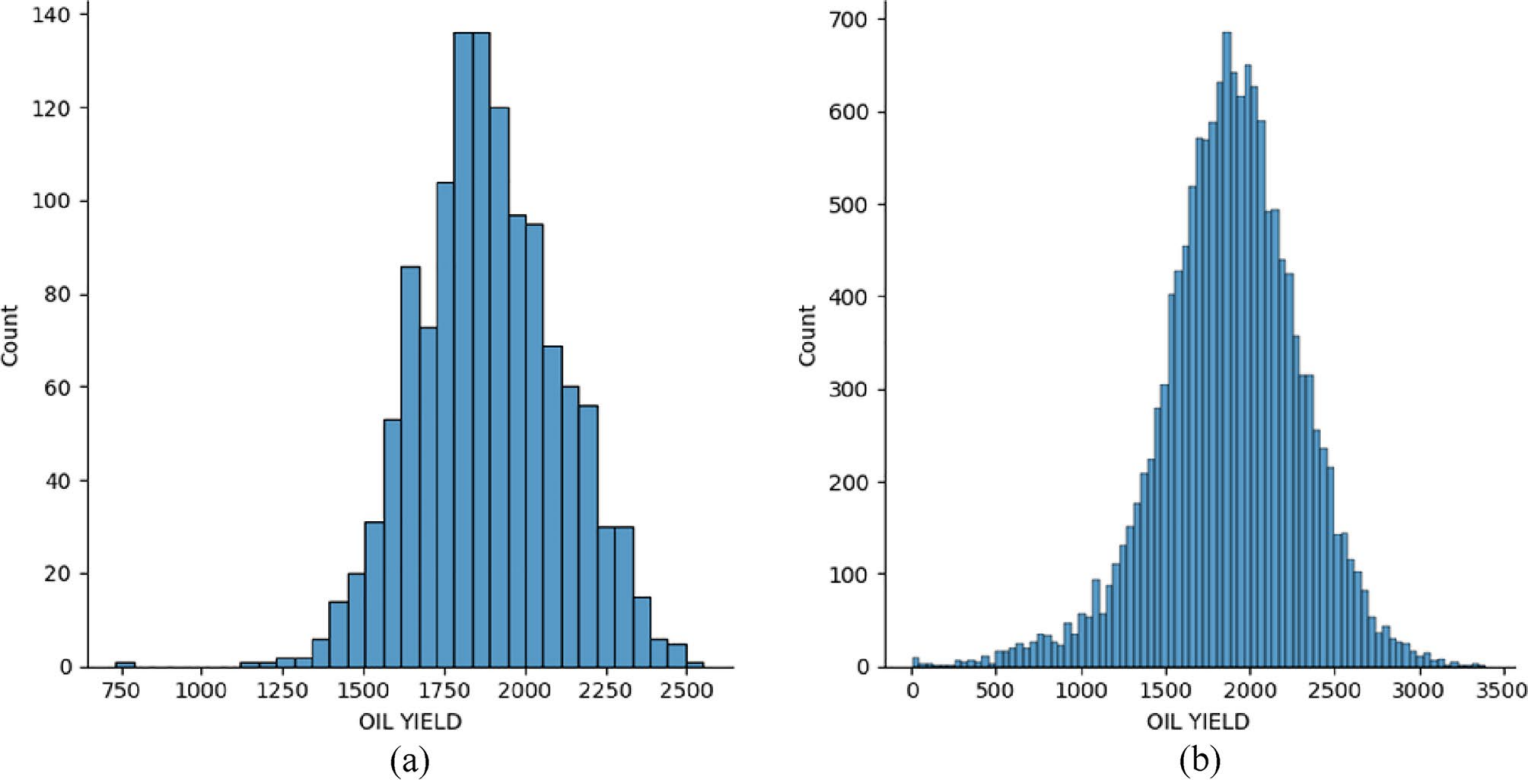
Target value (oil yield) distribution for the data from 2019 (**a**) first data subset (**b**) second data subset.

The target feature for both data subsets is the sunflower oil yield. For the first data subset, it is the oil yield for each hybrid (mean value from all 4 locations), and for the second data subset, it is the oil yield for each hybrid at each location. The distribution of the target feature is shown in Figs. 9 and 10.

**Figure 11:**
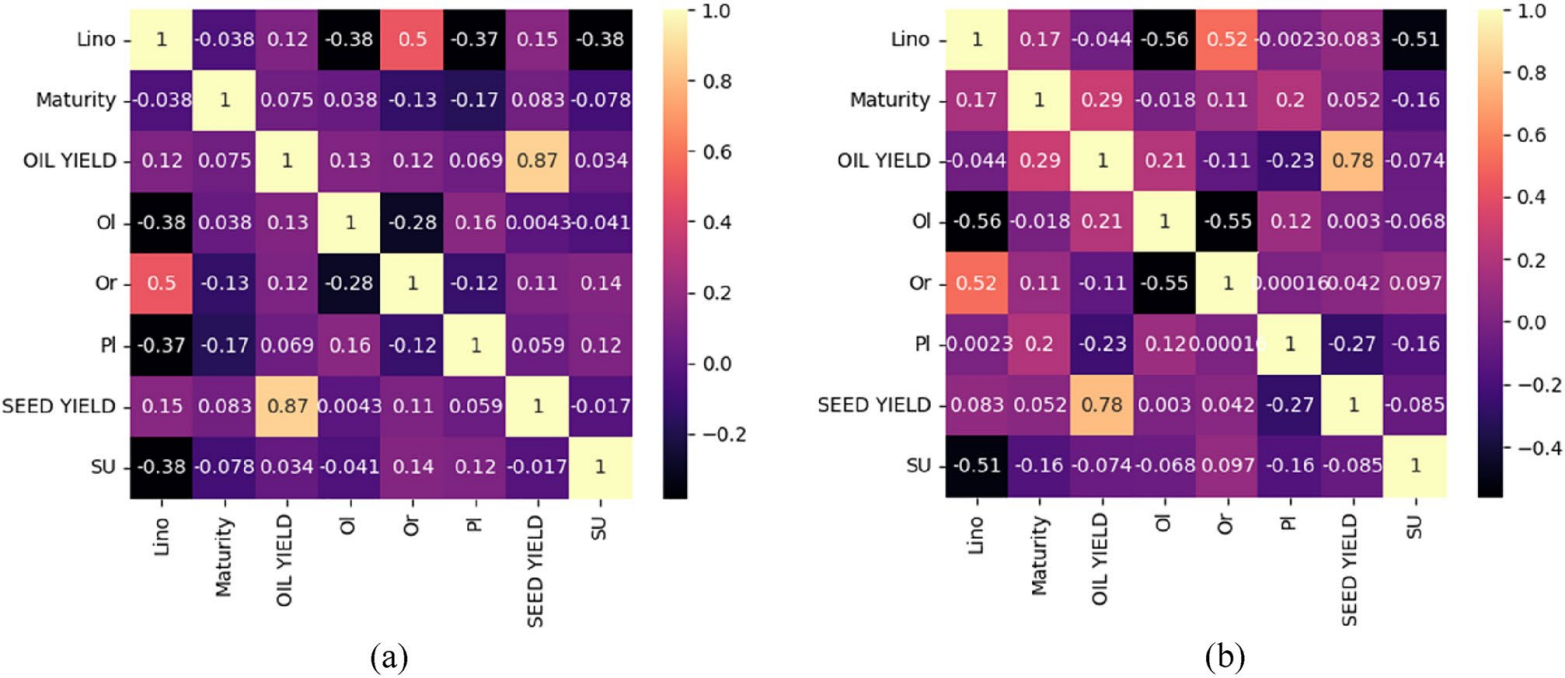
Pearson correlation coefficients of any two variables (**a**) 2018 (**b**) 2019.

Pearson correlation coefficients (PCCs) between every two variables are shown in Fig. 11. Correlations were calculated only for the first data subset because the second contained only categorical features. Both figures show a high correlation between the oil yield and the seed yield, which is expected. Oil yield was calculated based on seed yield and oil content. One of the goals of this work was to examine if hybrid characteristics or location characteristics can compensate for the lack of oil content data and thus predict it. Figure 11 shows that correlations between features are similar for both years. Since there are no features that are highly correlated with each other, we did not exclude any features from the initial data set.

### Algorithm description

#### Support vector regression

Support Vector Machine is a classification algorithm which tries to find a line or a hyperplane that divides classes. It classifies data points depending on whether it lies on the positive or negative side of the hyperplane. Support Vector Regression (SVR) is an algorithm which follows the same idea, but for the regression. SVR is widely used to forecast crop yields^35–37^. One of the advantages of this method is that mathematical analysis is more straightforward because nonlinear problems related to the input space are expressed pairing them with linear problems of high-dimension feature space^38^.

#### K-nearest neighbour

KNN is the supervised machine learning algorithm used for classification and regression. It has been used for predicting crop yield^37,39^. The algorithm manipulates the training data and classifies the new test data based on distance metrics. It finds a cluster of k samples that are nearest to unknown samples. From these k samples, the unknown samples are determined by calculating the average of the response variable. In KNN, k is the tuning parameter which plays a major role for an accurate prediction. The parameter of k is determined by running the model for values of k between 2 and 30 and finding the value which generates the highest R2. For the first data subsets the optimal *k* value was 12, and for the second data subset were 25, for both years. The algorithm could be considered beneficial in this study because it makes no assumptions about the data distribution.

#### Artificial neural network

ANN is a computational model which imitates the human brain. As an algorithm that can model complex relationships and patterns, the ANN is commonly applied to predict crop yield, often capturing subtle patterns that other algorithms may miss^32,37,40^. It has three types of layers: input layers, one or more hidden layers, and output layers. In this work, a feed-forward neural network with a backpropagation training algorithm is applied to find accurate crop yield. The number of input neurons differs based on feature sets which are obtained by using feature selection algorithms. The only tuning parameter of the algorithm is the number of hidden neurons which varies based on the number of input features. The optimizer that we used is Adam. For the first data subset from the 2018, optimal number of epochs was 20 and the batch size was 20. For the same data subset but from the 2019, optimal number of epochs was 30 and the batch size was 10. For the second data subset, the optimal number of epochs was 30 and 50 and the batch size was 10 and 20 for years 2018 and 2019 respectively. The number of hidden neurons that was used was 5.

#### Random forest regression

Random forest is a supervised ensemble machine learning algorithm that is widely used in classification and regression problems. Being an ensemble algorithm means it contains multiple decision trees. For regression tasks, the algorithm returns the mean or average prediction of the individual trees. The number of trees and the number of features in each split are needed as the input of the algorithm. RFR has been used in several articles to predict the crop yield^37,41,42^. RFR is renowned for its high accuracy, ability to handle large data sets with higher dimensionality, and its ability to handle missing values. It's also great for feature selection, as seen with the feature importance scores.

### Model evaluations

For model evaluations, we used mean absolute error (MAE), mean squared error (MSE), root mean square error (RMSE) and coefficient of determination (R2).

The absolute difference of the predicted value with the actual value defines the MAE, which is a measure of errors between paired observations expressing the same phenomenon (Eq. 1).

1 $$\mathrm{MAE }= \frac{\sum_{i=1}^{n}\left|{y}_{i}-{\widehat{y}}_{i}\right|}{n}$$

The MSE is the squared difference of the observed values of a variable with its predicted values, divided by the number of values for this variable (Eq. 2). It assesses the quality of the predictor.

2 $$\mathrm{MSE }= \frac{\sum_{i=1}^{n}{\left({y}_{i}-{\widehat{y}}_{i}\right)}^{2}}{n}$$

The RMSE is the square root of the MSE, indicating the standard deviation of the residuals (prediction errors) (Eq. 3).

3 $$\mathrm{RMSE }=\sqrt{ \frac{\sum_{i=1}^{n}{\left({y}_{i}-{\widehat{y}}_{i}\right)}^{2}}{n}}$$

The R2 is the proportion of the variance in the dependent variable which is explained by the linear regression model. The R2 will always be less than one (Eq. 4)

4 $$\mathrm{R}2 = 1 - \frac{\sum {\left({y}_{i}-{\widehat{y}}_{i}\right)}^{2}}{\sum {\left({y}_{i}-\overline{y }\right)}^{2}}$$

where, *n*—number of samples, *y*—actual oil yield value, $\widehat{y}$*—*predicted value of oil yield, $\overline{y }$—mean value of oil yield.

All analyses described here were performed using Python 3.6 (Python Software Foundation, Wilmington, DE, USA). For neural networks we used the Tensorflow framework (version 2.4.1) as backend and Keras (version 2.4.3) as frontend. The most significant libraries used are Pandas, sci-kit learn, Numpy, and Matplotlib (Supplementary Information S1).

### Supplementary Information

Supplementary Information.

## Data Availability

The datasets generated and/or analysed during the current study are not publicly available due to ongoing research that might be compromised by public disclosure at this stage. However, they are available from the corresponding author on reasonable request.
